# The overlooked diversity within a well-known diatom: Unraveling the *Luticola ventricosa* complex

**DOI:** 10.3897/phytokeys.274.190463

**Published:** 2026-05-11

**Authors:** Mateusz Rybak, Zlatko Levkov

**Affiliations:** 1 Faculty of Biotechnology, Medical College, University of Rzeszów, Ćwiklińskiej 2D, Rzeszów, Poland Faculty of Natural Sciences, Ss. Cyril and Methodius University Skopje Republic of North Macedonia; 2 Institute of Biology, Faculty of Natural Sciences, Ss. Cyril and Methodius University, Skopje, Republic of North Macedonia Medical College, University of Rzeszów Rzeszów Poland

**Keywords:** Bacillariophyceae, cryptic diversity, morphology, overlooked taxa, taxonomy

## Abstract

The *Luticola ventricosa* (Kützing) D.G.Mann has long been regarded as a morphologically variable and cosmopolitan species, yet its taxonomic concept has remained unstable due to the schematic original description. Recent revision of the type has provided a morphological framework, enabling critical reassessment of populations assigned to this species. During investigations focused on various populations originally treated as *L. ventricosa*, ten morphologically similar taxa were recognized within the complex. Four of these correspond to already described taxa (*L. ventricosa*, *L. dubia*, *L. levkovii*, *L. ventriconfusa*), while six taxa are described here as new to science: *L. borealis***sp. nov**., *L. exilissima***sp. nov**., *L. jagodae***sp. nov**., *L. lacunicola***sp. nov**., *L. microventricosa***sp. nov**., and *L. speluncae***sp. nov**. Results demonstrate that the diversity within the *L. ventricosa* complex has been substantially underestimated and that many earlier records of this species likely represent misidentifications. This study highlights the importance of detailed morphological revision, of even well-known species, for resolving cryptic diversity and improving understanding of species distributions and ecological differentiation.

## Introduction

The genus *Luticola* D.G.Mann (in Round et al. 1990, p. 670) was established to accommodate taxa previously included in the subgenus *Punctulata* (Grunow) Patrick of *Navicula* Bory, with *Luticola mutica* (Kützing) D.G.Mann (originally placed in genus *Navicula*) designated as the generitype. From its establishment in 1990 until the publication of its comprehensive monograph by Levkov et al. (2013), the genus received relatively little attention from diatom taxonomists. Since the publication of the monograph, *Luticola* has become a popular subject of taxonomic research, resulting in the description or transfer of over 80 taxa to the genus (Rybak et al. 2024a), and has similarly become an important subject of biogeographic studies (Kociolek et al. 2017). Today, the genus is relatively large, encompassing nearly 300 species distributed across all climatic zones (Rybak et al. 2024a; Guiry and Guiry 2026). Its representatives are known from a wide range of habitats, including freshwater and marine environments, but particularly from terrestrial ecosystems (Levkov et al. 2013; Zidarova et al. 2016). The considerable species richness is reflected in a wide morphological diversity within the genus. Nevertheless, certain features are consistent across all species: biraphid frustules, the presence of a bucciniportula in the central area with lipped internal opening, a thickened central area forming a stauros, narrow raphe-sternum, distinctly punctate striae, areolae present on valve mantle, mantle margin notched halfway between the apices and valve middle, open girdle bands (Rybak et al. 2024b), and a single chloroplast containing a single pyrenoid (Denys and De Smet 1996; Poulíčková 2008; Bagmet et al. 2023, 2024).

Among genus *Luticola* taxa with broadly capitate apices have been informally grouped within “Group M” sensu Levkov et al. (2013). The majority of these taxa are known from the Southern Hemisphere. Among taxa from Northern Hemisphere *L. ventricosa* (Kützing) D.G.Mann is one of the most commonly reported species. *Luticola ventricosa* represents a taxon with a particularly complex and long historical taxonomic background. Unlike the majority of species currently included in genus *Luticola*, which were originally described within *Navicula* and later transferred, *L. ventricosa* was first described as a member of genus *Stauroneis* (Kützing 1844, pl. 30: fig. 27). The original description was accompanied by only two highly schematic and poorly detailed illustrations, rendering the taxon difficult to interpret with certainty. The taxonomic history of this species, including its numerous transfers, has recently been comprehensively summarized by Van de Vijver et al. (2025) who also examined the type material. An additional uncertainty stems from the fact that type material was not examined during the preparation of the monograph of *Luticola* by Levkov et al. (2013). Although authors recognized morphological discontinuities among populations of *L. ventricosa*, they propose treating them as two separate morphotypes. Additionally, Levkov et al. (2013) also noted subtle, but consistent morphological differences between geographically separated populations of *L. ventricosa* may conceal distinct taxa. Beyond *L. ventricosa* sensu stricto, additional European species exhibiting subrostrate to capitate apices fall within the morphological framework traditionally associated with the *L. ventricosa* (complex). These include *L. bilyi* Levkov, Metzeltin & A.Pavlov, originally described from Kitzbühel (Tyrol, Austria) and also reported from the Slovak Tatra Mountains and the Jakupica in Macedonia (Levkov et al. 2013). As well as two species described after the publication of the genus monograph: *L. dubia* Levkov, Tofilovska, C.E.Wetzel, Mitic-Kopanja & Ector from halomorphic soils in the Ovče Pole, Macedonia and *L. levkovii* Reichardt from Bavaria, Germany and (Levkov et al. 2017; Reichardt 2018).

Recent observations show that the diversity of *L. ventricosa* complex is underestimated and there are still unknown or previously neglected. During the observations of various populations, 10 species similar to *L. ventricosa* were recorded, with six of them showing morphological characteristics different from known species and described herein as new based on LM and SEM observations.

## Material and methods

Samples from Poland and Turkey were prepared by boiling samples in 30% hydrogen peroxide (H_2_O_2_) for about three hours, while samples from Macedonia were cleaned by acid digestion, with the addition of 2 ml of K_2_MnO_4_ and 4 ml of HCl to a small (ca. 2 ml) subsample. To prepare permanent slides cleaned diatom material was pipetted onto coverslips and dried, then mounted on glass slides with Naphrax mounting medium (Brunel Microscopes Ltd, Wiltshire, UK).

Slide observations were performed with a Nikon Eclipse 80i (Nikon Corporation, Tokyo, Japan) microscope equipped with Differential Interference Contrast (DIC) under and a 100× Plan Apochromatic oil immersion objective (in case of all samples). For SEM (Scanning Electron Microscopy) analysis of Polish and Turkey samples, a few drops of material were placed on 5 µm pore size Whatman Nuclepore polycarbonate membrane filters (Fisher Scientific, Schwerte, Germany). Once air-dried in room temperature, the membranes were mounted on aluminium stubs and coated with 20 nm of gold using a turbo-pumped Quorum Q 150 T ES coater (Judges Scientific plc, London, UK). SEM observations were performed with a Hitachi microscope SU8010 (Hitachi Ltd, Tokyo, Japan).

For SEM observations of Macedonian samples, aliquots of the diatom sample were dried on a coverslip attached to stubs with carbon tape and coated with a thin gold-palladium layer (approximately 20 nm) using a Polaron SC7640 Sputter (Quorum Technologies, Ashford, UK). Scanning electron microscopy was performed at 5 kV and a 5 mm working distance using a Zeiss Gemini Ultra Plus FEM (Cambridge Instruments Ltd, Cambridge, UK).

Diatom terminology is used according to Round et al. (1990), Levkov et al. (2013) and Cox and Vijver (2024). The type slides are deposited at the Macedonian National Diatom Collection (**MKNDC**), Institute of Biology in Skopje, Macedonia and isotype slides are Szczecin Diatom Collection of the University of Szczecin, Poland (**SZCZ**). Additional material used in this study is deposited in the collection of the University of Rzeszów (**#**). All samples used in the study are listed below:

- MKDNC Acc. No. 002899 – North Macedonia, River Vardar, Near cave Bela Voda, epiphyton from *Cladophora*, coll. date: 09 March 2007, 41°24'19.0"N, 22°15'45.0"E;
- MKDNC Acc. No. 003083 – North Macedonia, Shara Mountain, pond below Dzinibeg, sediments, coll. date: 20 July 1995, 41°58'02.5"N, 20°46'09.6"E;
- MKDNC Acc. No. 004359 – North Macedonia, River Vardar, near village Jegunovce, rock scrape, coll. date: 17 October 2009, 42°04'31.5"N, 21°07'54.4"E;
- MKDNC Acc. No. 004431 – North Macedonia, Mountain Kitka, stream below cave Krasna, mosses, coll. date: 11 May 2002, 41°52'04.2"N, 21°29'52.4"E;
- MKDNC Acc. No. 007262 – North Macedonia, Mountain Korab, fen by the stream on Crn Kamen, wet mosses, coll. date: 10 July 2013, 41°49'47.8"N, 20°37'59.2"E;
- MKDNC Acc. No. 008839 – North Macedonia, Gladno Pole near Shtip, small peat bog, wet mosses, coll. date: 16 April 2015, 41°45'18.2"N, 22°09'29.3"E;
- MKDNC Acc. No. 011342 – North Macedonia, Mountain Kozuf, entrance of cave Temna, wet mosses, coll. date: 18 May 2013, 41°12'17.7"N, 22°00'10.0"E;
- MKDNC Acc. No. 011389 – North Macedonia, Mountain Stogovo, sand below snow field on Kenannica, coll. date: 12 June 2017, 41°29'40.6"N, 20°39'19.6"E;
- MKDNC Acc. No. 012659 – North Macedonia, Reservoir Paljurici near Bogdanci, sediments, coll. date: 13 February 2020, 41°12'24.8"N, 22°37'23.5"E;
- MKDNC Acc. No. 012861 – North Macedonia, Vitachevo plateau, fishpond Done Popov, sand, coll. date: 25 June 2020, 41°15'48.8"N, 22°03'15.7"E;
- MKDNC Acc. No. 012865 – North Macedonia, Vitachevo plateau, fishpond Done Popov, surface sediments, coll. date 25 June 2020, 41°15'48.8"N, 22°03'15.7"E;
- MKDNC Acc. No. 013814 – North Macedonia, Mountain Osogovo, fountain in monastery St. Pantelemon near vil. Pantelej, sediments, coll. date: 07 January 2021, 41°58'09.8"N, 22°18'37.5"E;
- MKDNC Acc. No. 013873 – North Macedonia, Mountain German, Ploce Litotelmi, wet mosses, coll. date 29 April 2022, 42°09'23.7"N, 22°01'25.1"E;
- MKDNC Acc. No. 014279 – North Macedonia, Mountain Osogovo, Vodena skala near village Trkanje, rock scrape, coll. date: 26 November 2022, 41°55'08.4"N, 22°21'37.1"E;
- MKDNC Acc. No. 015349 – North Macedonia, Lake Prespa, near Otehevo, sediments, coll. date 13 October 2025, 40°58'28.0"N, 20°54'59.0"E
- # 2016/1 – Poland, Stalowa Wola, permanent puddles on asphalt road, sediments, coll. date: 1 March 2016, 50°34'17.67"N, 22°04'33.78"E;
- # 2017/176 – Poland, Opatowiec, Vistula river, epiphyton, coll. date: 18 September 2017, 50°14'31.3"N, 20°43'38.0"E;
- # 2017/217 – Poland, Siadło Dolne, Oder river, sediments, coll. date: 11 October 2017, 53°20'22.7"N, 14°29'54.0"E;
- # 2019/72 – Turkey, near shore of Salda Lake, terrestrial mosses from soil, coll. date: November 2019, 37°33'28.6"N, 29°38'50.4"E;
- # 2019/73 – Turkey, near shore of Salda Lake, terrestrial mosses from calcareous rock, coll. date: November 2019, 37°33'28.6"N, 29°38'50.4"E;
- # 2019/95 – Poland, Nowiny, periodically flooded soil in a cranberry field, coll. date: September 2019, 50°42'06.9"N, 21°54'55.9"E;
- # 2025/119 – Poland, Bolestraszyce, terrestrial mosses from decaying log of *Robinia pseudoacacia* L., coll. date: 21 August 2025, 49°49'05.0"N, 22°51'39.6"E.

## Results

During the study 10 species which morphological correspond to *L. ventricosasensu stricto* were observed. Six species did not match any previously described *Luticola* taxa and are hereby described as new species. The morphological, geographical and ecological characteristics are described below. Size diminution series of the taxa were compiled to document as much as possible the variation in valve morphology throughout the life cycle.

### Observations of established taxa

#### Kingdom: ChromistaCavalier-Smith 1981

**Division: BacillariophytaHaeckel 1878**

**Class: BacillariophyceaeHaeckel 1878**

**Order: Mastogloiales Mann 1990**

**Family: Stauroneidaceae D.G.Mann 1990**

**Genus: *Luticola* D.G.Mann 1990**

##### Luticola ventricosa

Taxon classification

Plantae

NaviculalesDiadesmidaceae

(Kützing) D.G.Mann

D745C99A-7060-5F8C-8312-7064DF4A8D48

Fig. 1A–AM

###### Basionym.

*Stauroneis ventricosa* Kützing

**Figure 1.**
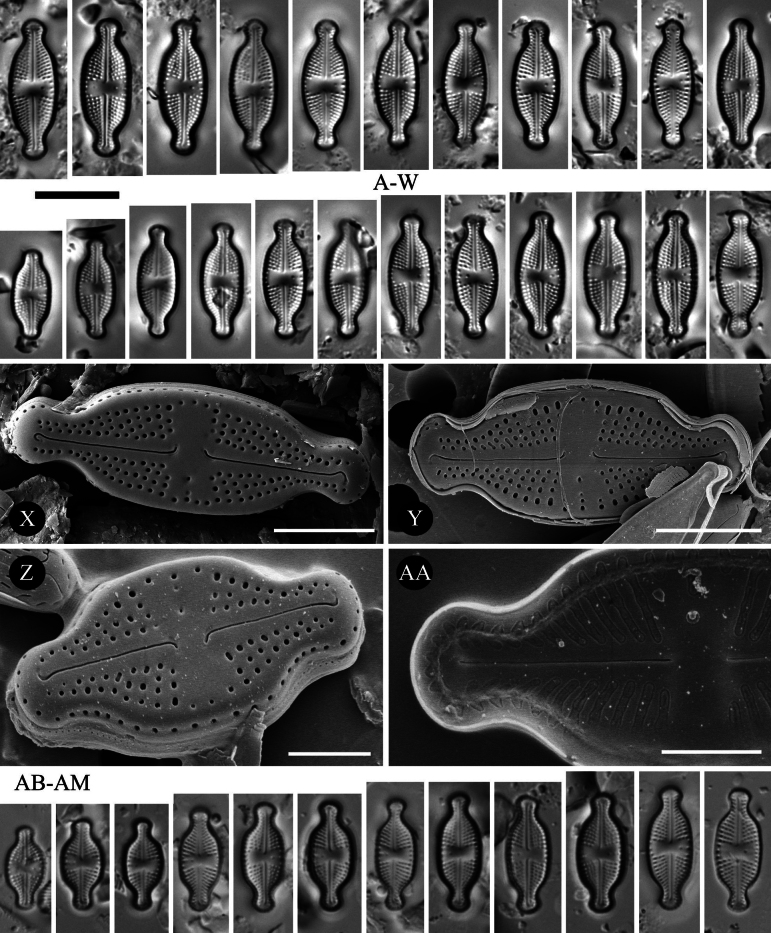
*Luticola ventricosa* (Kützing) D.G.Mann *sensu stricto* – size diminution series in light microscopy (**A–W, AB–AM**), SEM microphotographs of external (**X–Z**) and internal (**AA**) views. Specimens from Macedonia (**A–X**) (sample Acc. No. 13814), population from Poland (**Y–AM**) (sample 2025/114). Scale bars: 10 µm for LM; 5 µm (**X, Y**); 2.5 µm (**Z, AA**).

###### Synonyms.

*Navicula mutica* var. *ventricosa* (Kützing) Grunow (in Cleve and Grunow 1880: 41), *Navicula mutica* f. *ventricosa* (Kützing) Cleve (in Cleve 1894: 129), *Placoneis mutica* var. *ventricosa* (Kützing) Mereschkowsky (in Mereschkowsky 1903: 12), *Navicula ventricosa* (Kützing) F.W.Mills nom. illeg. (in Mills 1934: 1176), *Luticola mutica* var. *ventricosa* (Kützing) P.B.Hamilton (in Hamilton et al. 1994: 309), *Luticola mutica* f. *ventricosa* (Kützing) Bukhtiyarova (in Bukhtiyarova 1995: 421), *Navicula neoventricosa* Hustedt (in Hustedt 1966 [1966]: 612, fig. 1612 [first 4 illustrations]), (?) *Luticola aff. ventricosa* (Kützing) D.G.Mann (in Levkov et al. 2013, pl. 123, figs 34–39, pl. 128, figs 6–8).

###### Description (LM).

Valves lanceolate-elliptic with distinctly convex valve margins and protracted to capitate apices (Fig. 1A–W, AB–AM). Valve length 10.0–17.5 μm, valve width 4.5–6.5 μm. Striae in LM punctate, radiate throughout, 18–23 in 10 μm (Fig. 1A–W, AB–AMW). Transapical striae composed of usually 4 areolae. Central area asymmetrical, bowtie-shaped, bordered on both valve margins by 3–4 (usually 3) isolated areolae (Fig. 1A–W, AB–AM). Axial area very narrow, linear. Single isolated pore (bucciniportula) present in central area, located almost halfway between valve center and margin (Fig. 1A–W, AB–AM).

###### Description SEM.

Externally, bucciniportula with small, round opening (Fig. 1X–Z). Internally, bucciniportula with small opening bordered by narrow circular rim and covered with tongue-like structure (Fig. 1AA). Externally, raphe filiform, almost straight (Fig. 1X–Z). Proximal raphe endings bent opposite to bucciniportula with small depression around (Fig. 1X–Z). Distal raphe endings first weakly deflected opposite to pore-bearing side and then hooked towards pore-side, terminating on valve face (Fig. 1X–Z). Externally areolae are round to slightly elongate (Fig. 1X–Z). Striae at the apices composed by 2 areolae (Fig. 1X–Z). Internally, areolae occluded by hymenes forming continuous strip across valve (Fig. 1AA). Internally, raphe branches simple, linear (Fig. 1AA). Marginal channels located on valve face/mantle junction, narrow, indistinct and occluded with hymens (Fig. 1AA). Valve mantle with single row of round areolae (Fig. 1X, AA).

###### Ecology and distribution.

*Luticola ventricosa* is one of the most frequently recorded *Luticola* species, but some records might belong to other species (this study). In this study, *Luticola ventricosa* was observed in various subaerial habitats (on soils, mosses, and tree bark), as well as in small alpine ponds, where it occurred epiphytically on mosses or in surface sediments. Records from saline environments, including a saline spring and saline puddles observed in this study, further expand its known ecological range. However, its specific habitat preferences remain difficult to define.

##### Luticola dubia

Taxon classification

Plantae

NaviculalesDiadesmidaceae

Levkov, Tofilovska, C.E.Wetzel, Mitic-Kopanja & Ector

6B50D0E6-7693-5B32-B4A8-A1ED4AD729D8

Fig. 2A –AB

###### Description (LM).

**Figure 2.**
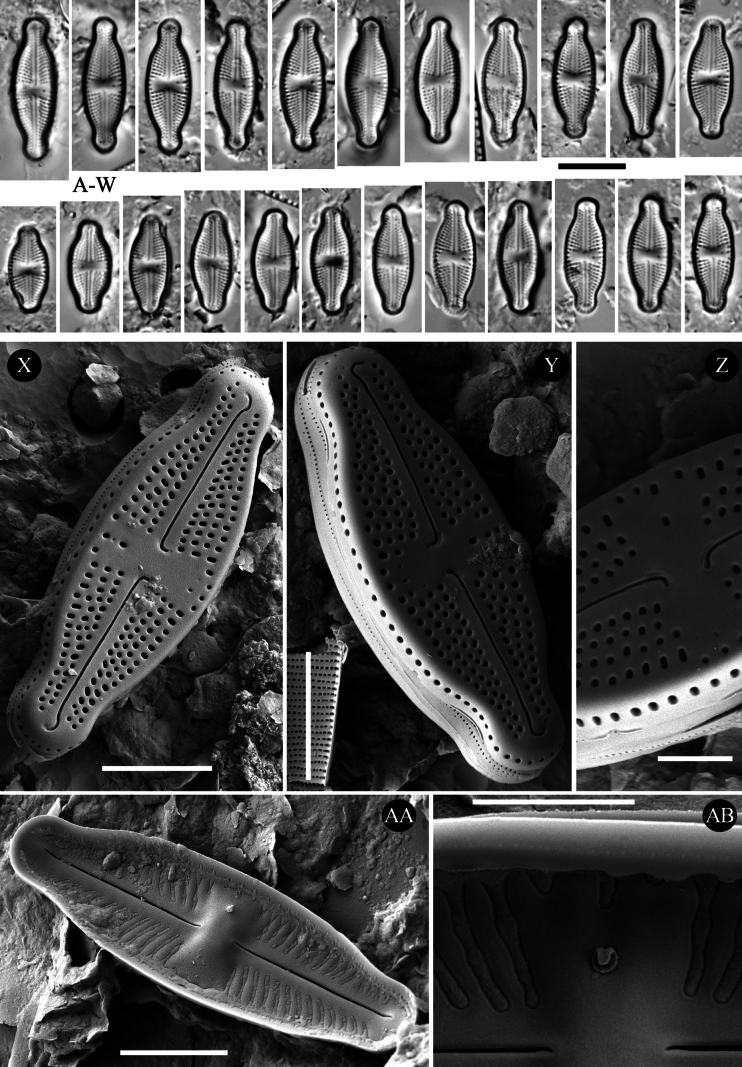
*Luticola dubia* Levkov, Tofilovska, C.E.Wetzel, Mitic-Kopanja & Ector – size diminution series in light microscopy (**A–W**), SEM microphotographs of external (**X–Z**) and internal (**AA, AB**) views. Scale bars: 10 µm for LM; 5 µm (**X, Y, AA**); 2 µm (**Z, AB**). Panels (X–AB) made by C.E. Wetzel & L. Ector.

Valves linear-lanceolate to narrowly elliptical with convex valve margins and narrowly protracted to subcapitate apices (Fig. 2A–W). Valve length 12.0–21.0 μm, valve width 5.0–6.5 μm. Striae in LM punctate, radiate throughout, 20–24 in 10 μm (Fig. 2A–W). Transapical striae composed of usually 4 areolae (Fig. 2A–W). Central area asymmetrical, bowtie-shaped, bordered on both valve margins by 3–4 (usually 3) isolated areolae. Axial area very narrow, linear (Fig. 2A–W). Single isolated pore present in central area, located almost halfway between valve center and margin (Fig. 2A–W).

###### Description SEM.

Externally, bucciniportula with small, round to slightly elongated opening (Fig. 2X–Z). Internally, bucciniportula with small opening bordered by narrow circular rim and covered with tongue-like structure (Fig. 2AB). Externally, raphe filiform and straight (Fig. 2X, Y). Proximal raphe endings bent opposite to bucciniportula with small depression around (Fig. 2X–Z). Distal raphe endings first weakly deflected opposite to pore-bearing side and then hooked towards pore-side, terminating on valve face (Fig. 2X–Z). Internally, raphe branches simple, linear (Fig. 2AA). Externally areolae are round to slightly elongate. Striae at the apices composed by 2 areolae (Fig. 2X–Z). Internally, areolae occluded by hymenes forming continuous strip across valve (Fig. 2AA, AB). Marginal channels located on valve face/mantle junction, narrow, indistinct and occluded with hymens (Fig. 2AA). Valve mantle with single row of round areolae (Fig. 2X–Z).

###### Ecology and distribution.

*Luticola dubia* has been observed only in halomorphic soils of Gladno Pole. The type locality comprises saline habitats in depressions, reed beds, and steppe-like communities on gentle slopes. The area is characterized by a very high groundwater level; during late winter and spring, small, dispersed springs are formed. In summer and autumn, the water level decreases, and salt precipitates on the soil surface (Levkov et al. 2017).

##### Luticola levkovii

Taxon classification

Plantae

NaviculalesDiadesmidaceae

E.Reichardt

FD7F00E8-518B-57C7-8651-3B4682114CBC

Fig. 3A–AG

###### Description LM.

**Figure 3.**
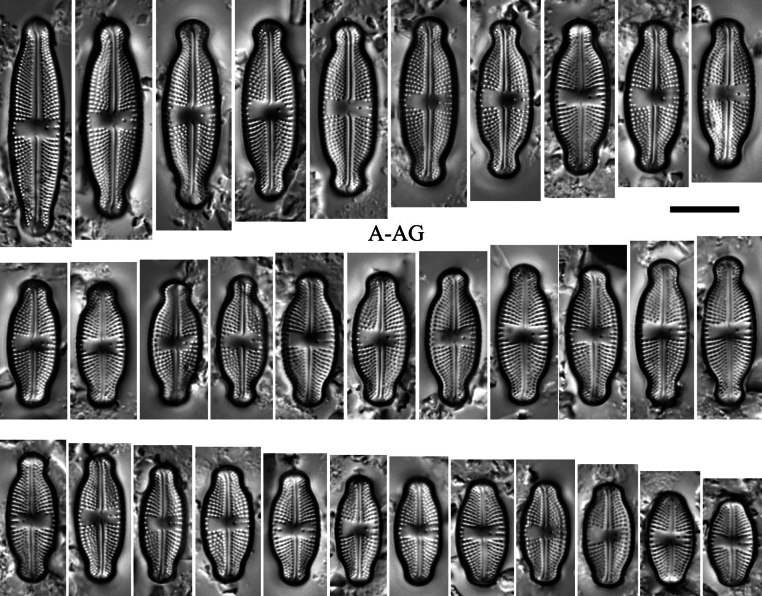
*Luticola levkovii* E.Reichardt. Size diminution series in light microscopy (**A–AG**). Scale bar: 10 µm.

Valves lanceolate-elliptic with convex valve margins and protracted to subcapitate apices (Fig. 3A–AG). Valve length 13.0–31.5 μm, valve width 5.8–7.8 μm. Striae in LM punctate, radiate throughout, 17–20 in 10 μm (Fig. 3A–AG). Transapical striae composed of usually (4)5–6 areolae (Fig. 3A–AG). Central area asymmetrical, rectangular to slightly bowtie-shaped, and bordered on both valve margins by 3–4 isolated areolae (Fig. 3A–AG). Axial area very narrow, linear (Fig. 3A–AG). Single isolated pore (bucciniportula) present in central area, located almost halfway between valve center and margin (Fig. 3A–AG). Raphe filiform with curved proximal endings and hooked, continuing onto valve mantle distal raphe endings (Fig. 3A–AG).

###### Ecology and distribution.

The type locality of *L. levkovii* is a small pond in Treuchtlingen, Germany (Reichardt 2018). In a similar habitat, *L. levkovii* was also observed in North Macedonia, in a small temporary pond formed by a spring on Osogovo Mountain (Levkov et al. 2025).

##### Luticola ventriconfusa

Taxon classification

Plantae

NaviculalesDiadesmidaceae

Lange-Bertalot

32BA223A-7334-53AC-80FB-71A4E09A4939

Fig. 4A–T

###### Description LM.

**Figure 4.**
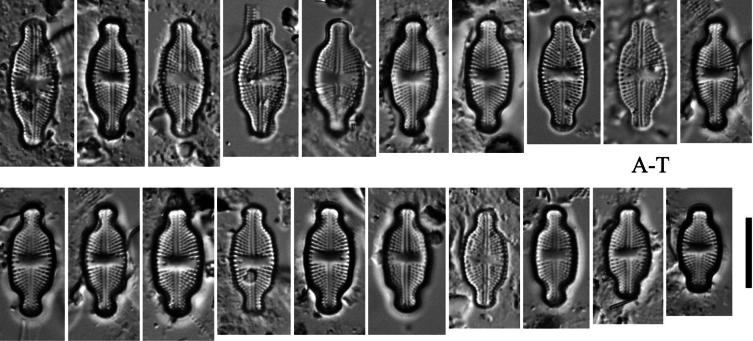
*Luticola ventriconfusa* Lange-Bertalot – size diminution series in light microscopy (**A–T**). Scale bar: 10 µm.

Valves linear-lanceolate, with weakly undulate margins (Fig. 4A–T). Valve margin undulations equally pronounced. Valve apices broadly rounded, subcapitate to rostrate in smaller specimens (Fig. 4A–T). Valve length 10.0–22.0 μm, valve width 5.8–6.8 μm. Striae in LM punctate, radiate throughout, 20–24 in 10 μm. Transapical striae composed of usually 4–5 areolae (Fig. 4A–T). Central area asymmetrical, rectangular to bowtie-shaped and bordered on both valve margins by usually 3 isolated areolae (Fig. 4A–T). Axial area very narrow, linear. Single isolated pore (bucciniportula) present in central area, located almost halfway between valve center and margin (Fig. 4A–T).

###### Ecology and distribution.

A species observed exclusively in samples collected from aquatic environments. It commonly occurs in rivers and lakes with moderate to high trophic levels e.g., the Vardar River, Lake Prespa (North Macedonia), and the Oder and Vistula rivers in Poland.

### New species description

#### Luticola borealis

Taxon classification

Plantae

NaviculalesDiadesmidaceae

M.Rybak & Levkov sp. nov.

5C896579-C963-5E5C-A12E-8904F8E7FACE

Fig. 5A–W

##### Synonym.

*Luticola ventricosa* MT1 (Kützing) D.G.Mann (in Levkov et al. 2013, pl. 123, figs: 1–25, pl. 124, figs: 1–37).

**Figure 5.**
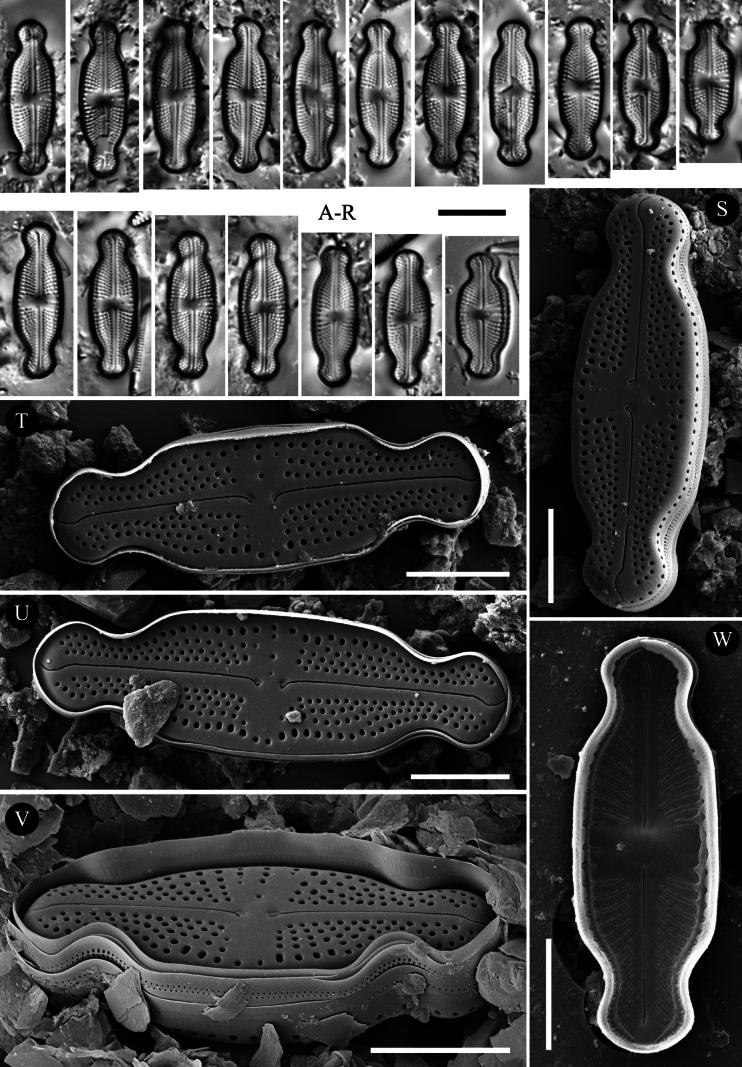
*Luticola borealis* sp. nov. – size diminution series in light microscopy (**A–R**), SEM microphotographs of external (**S–V**) and internal (**W**) views. Specimens from Macedonia (**A–U**) (sample Acc. No. MKNDC 013873), population from Poland (**V, W**) (sample 2016/8). Scale bars:10 µm for LM; 5 µm (**S–W**).

##### Description LM.

Valves lanceolate-elliptic with parallel valve margins and protracted to subcapitate apices (Fig. 5A–R). Valve dimensions (n = 100): length (9.0)12.5–28.0 μm, width 6.0–8.5 μm. Striae in LM punctate, radiate throughout, 16–21 in 10 μm (Fig. 5A–R). Transapical striae composed of usually 4 areolae (Fig. 5A–R). Central area asymmetrical, rectangular to slightly bowtie-shaped and bordered on both valve margins by 3–4 (usually 3) isolated areolae (Fig. 5A–R). Axial area very narrow, linear (Fig. 5A–R). Single isolated pore (bucciniportula) present in central area, located almost halfway between valve center and margin (Fig. 5A–R).

##### Description SEM.

Externally, bucciniportula with small, round opening (Fig. 5S–V). Internally, bucciniportula with small opening bordered by narrow circular rim and covered with tongue-like structure (Fig. 5W). Externally, raphe filiform and straight (Fig. 5S–U). Proximal raphe endings bent opposite to bucciniportula with small depression around (Fig. 5S–V). Distal raphe endings prolonged, first slightly curved towards same side as proximal endings then deflected towards opposite side, continuing onto valve mantle (Fig. 5S–U). Internally, raphe branches simple, linear. Externally areolae are round to slightly elongate. Striae at the apices composed by 2–3 areolae (Fig. 5S–U). Internally, areolae occluded by hymenes forming continuous strip across valve (Fig. 5W). Marginal channels located on valve face/mantle junction, narrow, indistinct and occluded with hymens, interrupted at apices (Fig. 5W). Valve mantle with single row of round areolae (Fig. 5S). Girdle bands with two rows of areolae (Fig. 5V).

##### Type.

Republic of North Macedonia • wet mosses in pond, Ploce Litotelmi, Mountain German, 42°09'23.7"N, 22°01'25.1"E, leg. Z. Levkov, coll. date: 29.04.2022, ***Holotype***: Acc. No. MKNDC 013873, ***Isotype***: SZCZ 29719.

***Paratype 1***: • sand, Fish pond Done Popov, Mountain Kozuf, 41°15'48.8"N, 22°03'15.7"E, leg. Z. Levkov, coll. date: 25.06.2020, Acc. No. MKNDC 012861.

***Paratype 2***: • sediments in shallow puddle, Podkarpacie Province, Stalowa Wola, 50°34'17.67"N, 22°04'33.78"E, M. Rybak, 12.11.2016, sample RZ 2016/1.

##### Phycobank.

http://phycobank.org/107067.

##### Etymology.

The epithet ‘borealis’ refers to the predominantly northern distribution of the species.

##### Ecology and distribution.

A widely distributed species, recorded from stagnant waters (reservoirs, lakes, puddles), as well as from peatbogs and terrestrial environments (see Discussion).

#### Luticola exilissima

Taxon classification

Plantae

NaviculalesDiadesmidaceae

Levkov & M.Rybak sp. nov.

3E386B04-6C54-5B59-8B7B-09932FABC932

Fig. 6A–Y

##### Description (LM).

**Figure 6.**
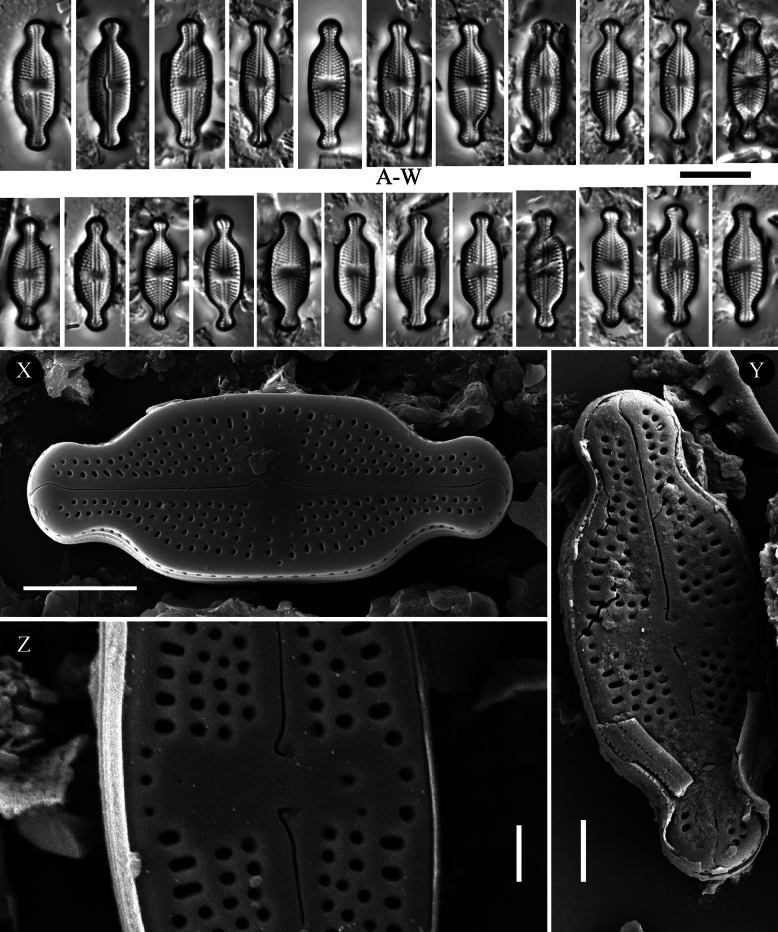
*Luticola exilissima* sp. nov. – size diminution series in light microscopy (**A–W**), SEM microphotographs of external (**X–Z**) views. Scale bars: 10 µm for LM; 5 µm (**X**); 2 µm (**Y**); 1 µm (**Z**).

Valves linear-elliptic with parallel valve margins and protracted to capitate apices (Fig. 6A–W). Valve dimensions (n = 30): length 15.0–18.5 μm, width 5.0–6.0 μm. Striae in LM punctate, radiate throughout, 20–22 in 10 μm (Fig. 6A–W). Transapical striae composed of 3–4 areolae (Fig. 6A–W). Central area asymmetrical, rectangular to bowtie-shaped, bordered on both valve margins by 3–4 isolated areolae (Fig. 6A–W). Axial area very narrow, linear. Single isolated pore (bucciniportula) present in central area, located almost halfway between valve center and margin (Fig. 6A–W).

##### Description SEM.

Externally, bucciniportula with small, round opening (Fig. 6X–Z). Externally, raphe filiform and straight (Fig. 6X–Z). Proximal raphe endings tiny, sharply hooked to bucciniportula site with small depression around (Fig. 6X–Z). Distal raphe endings prolonged, first slightly curved towards same side as proximal endings then deflected towards opposite side, continuing onto valve mantle (Fig. 6X–Z). Externally areolae are round to slightly elongate (Fig. 6X–Z). Striae at the apices composed by 2 areolae (Fig. 6X–Z). Valve mantle with single row of areolae.

##### Type.

Republic of North Macedonia • wet rock at Vodna Skala, Frcelo near vil. Trkanje, Mountain Osogovo, 41°55'08.4"N, 22°21'37.1"E, leg: D. Zaova, coll. date: 26.11.2022, ***Holotype***: Acc. No. MKNDC 014279, ***Isotype***: SZCZ 29720.

##### Phycobank.

http://phycobank.org/107068.

##### Etymology.

The specific epithet (exilissima) refers to narrower (thin) valves of the species.

##### Ecology.

Species was observed in subaerial habitats, particularly on wet silicate rocks.

#### Luticola jagodae

Taxon classification

Plantae

NaviculalesDiadesmidaceae

M.Rybak, Solak & Levkov sp. nov.

3E3DD6C2-A107-5E45-9A6B-D6BD28DB556E

Fig. 7A–Z

##### Description (LM).

**Figure 7.**
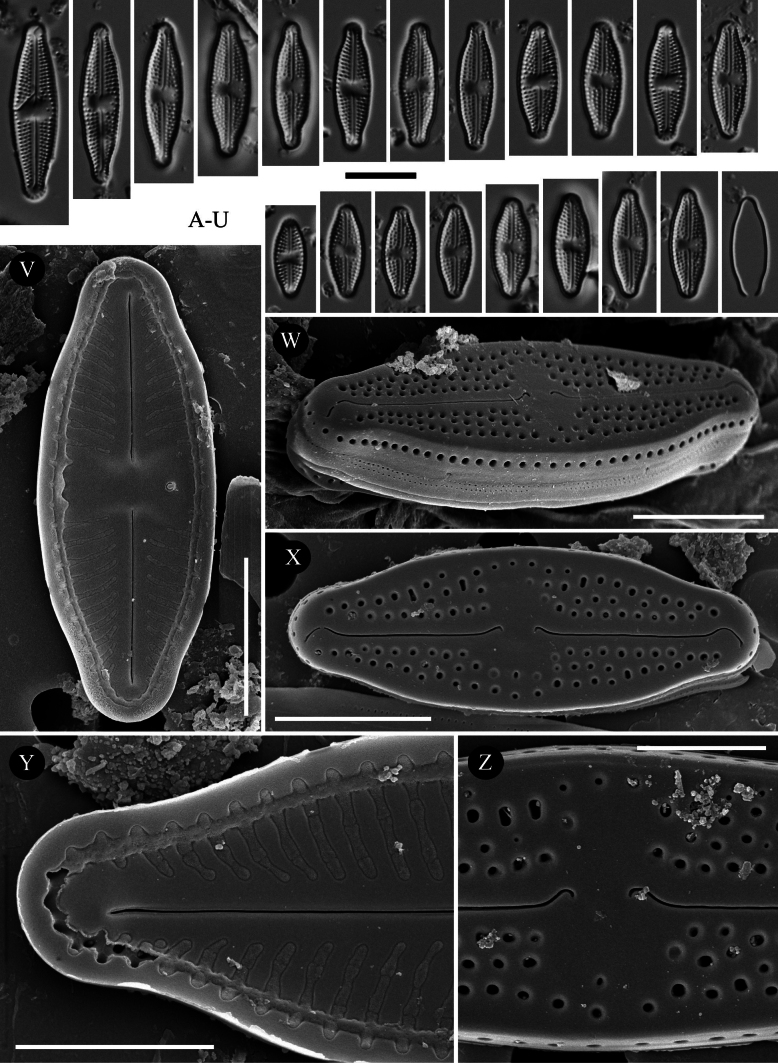
*Luticola jagodae* sp. nov. – size diminution series in light microscopy (**A–U**), SEM microphotographs of external (**W, X, Z**) and internal (**V, Y**) views. Scale bars: 10 µm for LM; 5 µm (**V, W, X**); 3 µm (**Y**); 2.5 µm (**Z**).

Valves narrow, linear-lanceolate with convex valve margins and protracted apices (Fig. 7A–T). Valve dimensions (n = 50): length 10.0–25.0 μm, width 4.0–6.0 μm. Striae in LM punctate, radiate throughout, 17–21 in 10 μm (Fig. 7A–T). Transapical striae composed of usually 3 areolae (Fig. 7A–T). Central area rectangular to slightly bowtie-shaped, bordered on both valve margins by 3–4 isolated areolae (Fig. 7A–T). Axial area very narrow, linear (Fig. 7A–T). Single isolated pore (bucciniportula) present in central area, located almost halfway between valve center and margin (Fig. 7A–T). Girdle bands open (Fig. 7U).

##### Description SEM.

Externally, bucciniportula with small, round opening (Fig. 7W, X, Z). Internally, bucciniportula with small opening bordered by narrow circular rim and covered with tongue-like structure (Fig. 7V, Y). Externally, raphe filiform and straight (Fig. 7W, X). Proximal raphe endings curved towards same side as proximal endings then deflected towards opposite side (Fig. 7W, X, Z). Distal raphe endings prolonged, first slightly curved towards same side as proximal endings then deflected towards opposite side, terminating on valve face/mantle junction (Fig. 7W, X). Internally, raphe branches simple, linear (Fig. 7V, Y). Externally areolae both on valve face and valve mantle are round (Fig. 7W, X). Striae at the apices composed by 1–2 areolae (Fig. 7W, X). Internally, areolae occluded by hymenes forming continuous strip across valve (Fig. 7V, Y). Marginal channels located on valve face/mantle junction, and occluded with hymens (Fig. 7V, Y). Valve mantle with single row of round areolae (Fig. 7W). Girdle bands with 2 rows of round pores (Fig. 7W).

##### Type.

***Holotype***: Republic of Türkiye • Yeşilova district, Burdur Province, 37°33'28.6"N, 29°38'50.4"E, moss sample from Edge of the coniferous forest near Lake Salda shore, leg. M. Rybak and C.N. Solak, November 2019, ***Holotype***: MKNDC 015446, ***Isotype***: SZCZ 29721.

##### Phycobank.

http://phycobank.org/107069.

##### Etymology.

The species is dedicated to prof. Jadwiga “Jagoda” Stanek-Tarkowska in recognition of initiating studies on soil diatoms at the University of Rzeszów.

##### Ecology and distribution.

Terrestrial species, observed in two samples of plagiotropic mosses – one from limestone rock and one from soil.

#### Luticola lacunicola

Taxon classification

Plantae

NaviculalesDiadesmidaceae

Levkov & M.Rybak sp. nov.

BC2C1881-D3C3-5C2B-946A-E9095A7DEC2B

Fig. 8A–AB

##### Synonym.

*Luticola ventricosa* (Kützing) D.G.Mann MT2 (in Levkov et al. 2013, pl. 125, figs 1–6).

**Figure 8.**
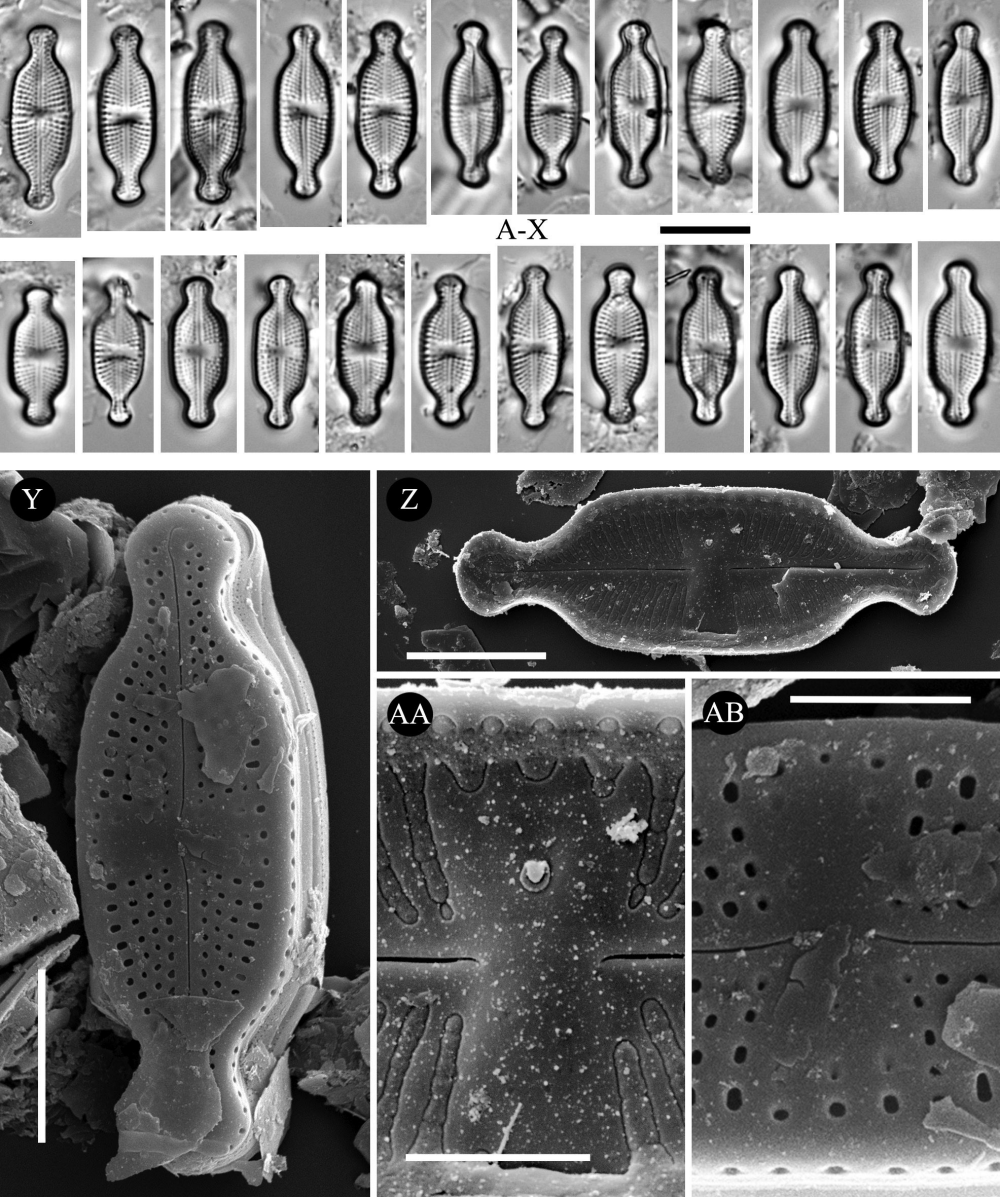
*Luticola lacunicola* sp. nov. – size diminution series in light microscopy (**A–X**), SEM microphotographs of external (**Y, AB**) and internal (**Z, AA**) views. Scale bars: 10 µm for LM; 5 µm (**Y, Z**); 2 µm (**AA, AB**).

##### Description (LM).

Valves linear-elliptic with parallel valve margins and protracted to capitate apices (Fig. 8A–X). Valve dimensions (n = 30): length 15.0–19.0 μm, width 5.5–6.0 μm. Striae in LM punctate, radiate throughout, 18–22 in 10 μm (Fig. 8A–X). Transapical striae composed of usually 4–5 areolae (Fig. 8A–X). Central area small and asymmetrical, rhombic to bowtie-shaped, bordered on both valve margins by 3–4 shortened striae or isolated areolae (Fig. 8A–X). Axial area very narrow, linear. Single isolated pore (bucciniportula) present in central area, slightly shifted to valve center (Fig. 8A–X).

##### Description SEM.

Internally, bucciniportula with small opening bordered by narrow circular rim and covered with tongue-like structure (Fig. 8Z, AA). Externally, raphe filiform and straight (Fig. 8Y). Proximal raphe endings tiny, almost straight (Fig. 8AB). Distal raphe endings strongly bent to bucciniportula site, terminating on valve face (Fig. 8Y). Internally, raphe branches simple, linear (Fig. 8Z). Externally areolae are round to slightly elongate (Fig. 8Y). Striae at the apices composed by 2 areolae (Fig. 8Y). Internally, areolae occluded by hymenes forming continuous strip across valve (Fig. 8Z). Marginal channels located on valve face/mantle junction, narrow, indistinct and occluded with hymenes (Fig. 8Z). Valve mantle with single row of round areolae (Fig. 8Y).

##### Type.

Republic of North Macedonia • sediment in alpine pond below Dzinibeg, Shara Mountain, 41°58'02.5"N, 20°46'09.6"E, leg. Z. Levkov, coll. date: 20.07.1995, ***Holotype***: Acc. No. MKNDC 003083, ***Isotype***: SZCZ 29722.

##### Phycobank.

http://phycobank.org/107070.

##### Etymology.

The specific epithet (lacunicola) refers to the habitat pond (*lacus*) where this species was observed (*cola*).

##### Ecology and distribution.

Freshwater oligotrophic species occurring in alpine ponds. So far it has been observed in a few alpine ponds on Shara Mountain, North Macedonia.

#### Luticola microventricosa

Taxon classification

Plantae

NaviculalesDiadesmidaceae

Levkov & M.Rybak sp. nov.

6A2B3E09-79D8-5F5F-9E5F-2871FD0E2F5E

Fig. 9A –AO

##### Description (LM).

**Figure 9.**
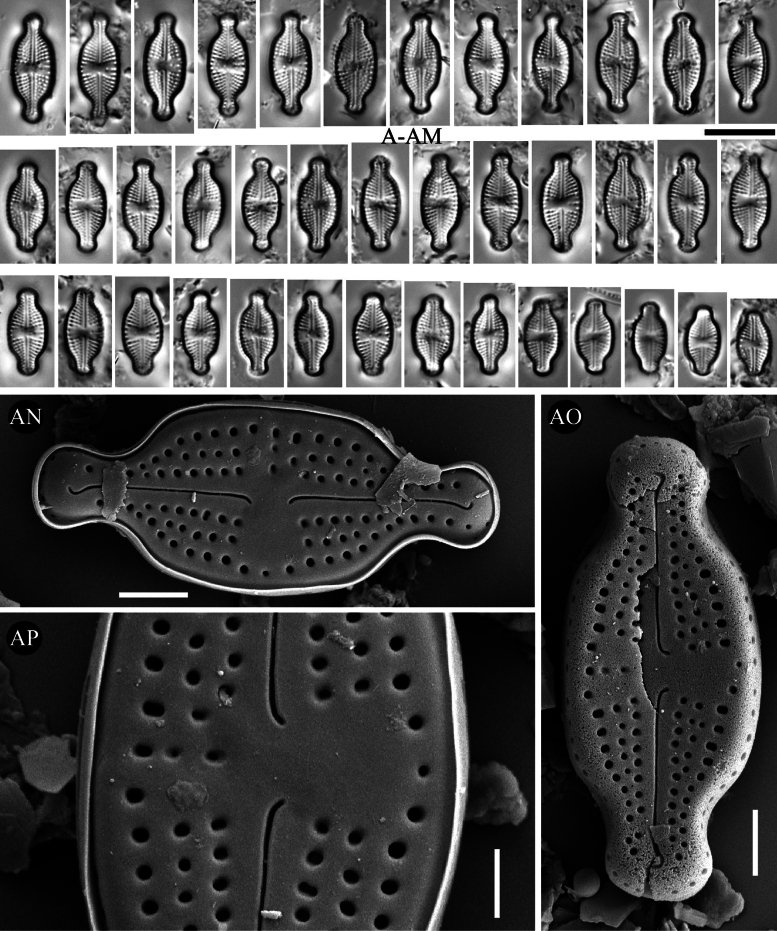
*Luticola microventricosa* sp. nov. – size diminution series in light microscopy (**A–AM**), SEM microphotographs of external (**AN–AP**) views. Scale bars: 10 µm for LM; 2 µm (**AN, AO**); 1 µm (**AP**).

Valves elliptic with convex valve margins and protracted to subcapitate apices (Fig. 9A–AM). Valve dimensions (n = 30): length 9.0–15.0 μm, width 4.5–5.5 μm. Striae in LM punctate, radiate throughout, 16–18 in 10 μm. Transapical striae composed of usually 3 areolae (Fig. 9A–AM). Central area asymmetrical, bowtie-shaped, bordered on both valve margins by 3 (rarely 2) isolated areolae (Fig. 9A–AM). Axial area very narrow, linear. Single isolated pore (bucciniportula) present in central area, located almost halfway between valve center and margin (Fig. 9A–AM).

##### Description SEM.

Externally, bucciniportula with small, round opening (Fig. 9AP). Externally, raphe filiform and straight (Fig. 9AN, AO). Proximal raphe endings slightly bent opposite to bucciniportula (Fig. 9AN). Distal raphe endings first weakly deflected opposite to pore-bearing side and then hooked towards pore-side, terminating on valve face (Fig. 9AN, AO). Externally areolae are round (Fig. 9AN, AO). Striae at the apices composed mainly by single areolae (Fig. 9AN). Valve mantle with single row of round areolae (Fig. 9AO).

##### Type.

Republic of North Macedonia • wet moss near river Crn Kamen, Mountain Korab, 41°49'47.8"N, 20°37'59.2"E, leg: Z. Levkov, coll. date: 10.07.2013, ***Holotype***: Acc. No. MKNDC 007262, ***Isotype***: SZCZ 29723

##### Phycobank.

http://phycobank.org/107071.

##### Etymology.

The specific epithet (microventricosa) refers to the smaller valve of the species compared to the *L. ventricosa*.

##### Ecology.

The type locality is a subaerial wet moss site near a small mountain stream on karstic substrate in alpine grassland.

#### Luticola speluncae

Taxon classification

Plantae

NaviculalesDiadesmidaceae

Levkov & M.Rybak sp. nov.

8100187E-52DD-5EEF-A8A7-CA7787FC273C

Fig. 10A–AL

##### Description (LM).

**Figure 10.**
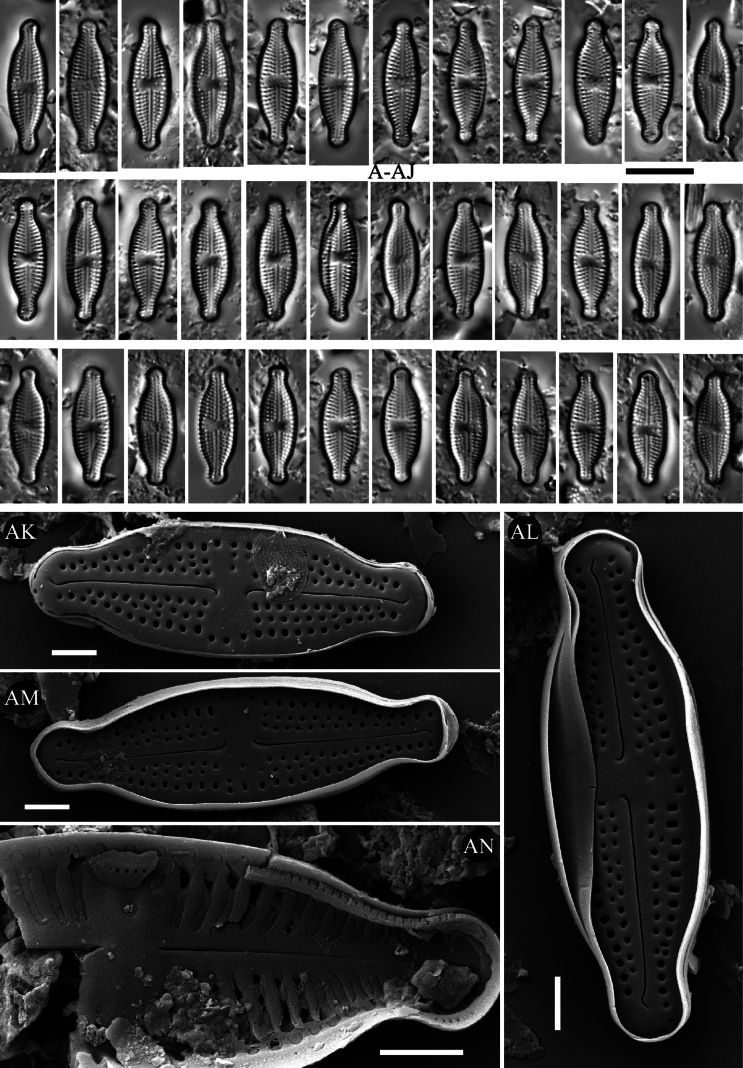
*Luticola speluncae* sp. nov. – size diminution series in light microscopy (**A–AJ**), SEM microphotographs of external (**AK–AM**) and internal (**AN**) views. Scale bars: 10 µm for LM; 2 µm (**AK–AN**).

Valves narrow, linear-lanceolate with convex valve margins and protracted to subcapitate apices (Fig. 10A–AJ). Valve dimensions (n = 30): length 16.0–20.0 μm, valve width 5.0–5.5 μm. Striae in LM punctate, radiate throughout, 16–18 in 10 μm (Fig. 10A–AJ). Transapical striae composed of usually 3 areolae (Fig. 10A–AJ). Central area asymmetrical, rectangular to slightly bowtie-shaped, bordered on both valve margins by usually 3 isolated areolae (Fig. 10A–AJ). Axial area very narrow, linear. Single isolated pore (bucciniportula) present in central area, located almost halfway between valve center and margin (Fig. 10A–AJ).

##### Description SEM.

Externally, bucciniportula with small, round opening (Fig. 10AK–AL). Internally, bucciniportula with small opening bordered by narrow circular rim and covered with tongue-like structure (Fig. 10AN). Externally, raphe filiform, straight to slightly curved (Fig. 10AK–AM). Proximal raphe endings bent opposite to bucciniportula with small depression around (Fig. 10AK–AM). Distal raphe endings first weakly deflected opposite to pore-bearing side and then slightly deflected towards pore-side, terminating on valve face (Fig. 10AK–AL). Internally, raphe branches simple, linear (Fig. 10AN). Externally areolae are round to elongate along valve margin (Fig. 10AK–AM). Striae at the apices composed by 1–2 areolae (Fig. 10AK–AM). Internally, areolae occluded by hymenes forming continuous strip across valve (Fig. 10AN). Marginal channels located on valve face/mantle junction, narrow, indistinct and occluded with hymenes (Fig. 10AN). Girdle bands with scattered pores (Fig. 10AL).

##### Type.

Republic of North Macedonia • wet mosses at entrance of the Temna Cave near vill. Mrezicko, Mountain Kozuf, 41°12'17.7"N, 22°00'10.0"E, leg. Z. Levkov, coll. date: 18.03.2013, ***Holotype***: Acc. No. MKNDC 011342, ***Isotype***: SZCZ 29724

##### Phycobank.

http://phycobank.org/107072.

##### Etymology.

The specific epithet (speluncae) refers to habitat – entrance of the cave (Latin: *spelunca* = cave).

##### Ecology.

The new species was observed on wet rock at entrance of the cave. It is a typical subaerial habitat that becomes completely dry during the late summer and early autumn.

## Discussion

The taxonomic concept of *Luticola ventricosa* has long been affected by instability and ambiguity, resulting from limited detail in the original description (Kützing 1844) and the absence of a comprehensive analysis of the type material for monography of the genus *Luticola*, combined with the description of morphologically similar taxa (Levkov et al. 2013). The lack of a clearly defined reference point has likely contributed to an oversimplified understanding of the species’ distribution, which is currently regarded as cosmopolitan, with records reported from all continents (Guiry and Guiry 2026). The recent, detailed revision of the type material (Van de Vijver et al. 2025) provided a morphological reference framework, enabling reliable comparative analyses among populations from different habitats and regions. As demonstrated in the present study, this refinement of the typological concept was crucial for recognizing consistent morphological differences among populations previously assigned to *L. ventricosa*, ultimately leading to the description of few previously overlooked but distinct species. Based on the documentation of the type material, *L. ventricosa* appears very likely to be conspecific with *L. aff. ventricosa* presented in Levkov et al. (2013, pl. 123, figs 34–39, pl. 128, figs 6–8).

Described from halomorphic soils, *L. dubia* represents the morphologically closest taxon to *L. ventricosasensu stricto*. Both taxa share very similar raphe structure with hooked distal endings which terminate on the valve face, and the proximal raphe endings distinctly deflected and accompanied by small depressions. Additionally, both taxa can be distinguished based on the valve outline: *L. dubia* exhibits a distinctly linear-lanceolate shape (Levkov et al. 2017), clearly different from the broader, more elliptic outline of *L. ventricosa* (Van de Vijver et al. 2025).

*Luticola microventricosa* sp. nov. can be distinguished from *L. ventricosasensu stricto* by several consistent morphological characters. The new species is smaller, measuring 9–15 µm in length and 4.5–5.5 µm in width, compared to 10.0–17.5 µm and 5.5–6.5 µm, respectively, in *L. ventricosa*. Stria density is lower in *L. microventricosa* sp. nov. (16–18 in 10 µm versus 18–22 in *L. ventricosa*), and each stria consistently contains three areolae, whereas *L. ventricosa* exhibits 3–4 areolae per stria. The distal raphe endings are similar in both species, terminating on the valve face, but the proximal raphe endings in *L. microventricosa* sp. nov. are only slightly deflected. Additionally, striae at the apices of *L. microventricosa* sp. nov. always consist of a single areola, in contrast to the generally two areolae observed in *L. ventricosa* (Van de Vijver et al. 2025). The striae at the apices, which consist of single areolae, represent a unique feature of this taxon, not observed in other European species within the complex. European *Luticola microventricosa* sp. nov is also very similar to *L. dolia* Spauldin & Esposito from Antarctica. Both species share a low number of areolae per stria, including single areolae at the apices, and a comparable structure of the proximal raphe endings (Esposito et al. 2008). Despite these similarities, the two species are clearly distinguishable based on their size: *L. microventricosa* sp. nov. measures 9.0–15.0 µm in length and 5.5–6.5 µm in width, whereas *L. dolia* is clearly larger, ranging from 15.0–22.0 µm in length and 5.5–8.0 µm in width (Esposito et al. 2008).

*Luticola speluncae* sp. nov. and *L. jagodae* sp. nov shows great degree of similarity, however both can be easily separated based on their dimensions as well as based on the structure of both raphe endings. The valve dimensions of *L. speluncae* sp. nov. are narrower and more restricted (16.0–20.0 µm in length, 5.0–5.5 µm in width) compared to *L. jagodae* sp. nov., which ranges from 10.0–25.0 µm in length and 4.0–6.0 µm in width. Stria density also differs slightly, with *L. speluncae* sp. nov. exhibiting 16–18 striae in 10 µm versus 17–21 in *L. jagodae* sp. nov. The proximal raphe endings are deflected in *L. speluncae* sp. nov., whereas in *L. jagodae* sp. nov. they are clearly hooked. Distal raphe endings are deflected and terminate on the valve face in *L. speluncae* sp. nov., in contrast to *L. jagodae* sp. nov., where they are hooked and terminate at the valve face-mantle junction. Moreover, *Luticola speluncae* sp. nov. exhibits more elongated and distinctly differentiated valve apices compared to *L. jagodae* sp. nov.

Despite generally overlapping valve dimensions between *Luticola exilissima* sp. nov. and *L. ventricosasensu stricto* (see Table 1) both species can be easily separated based on the valve outline. Valve margin in *L. exilissima* sp. nov. exhibits clearly parallel margins, whereas *L. ventricosa* has convex margins (Van de Vijver et al. 2025). Proximal raphe endings in *L. exilissima* sp. nov. are tiny, sharply hooked, and accompanied by a small depression, contrasting with the clearly bent proximal endings of *L. ventricosa* (Van de Vijver et al. 2025). Distal raphe endings are hooked in both species, but in *L. exilissima* sp. nov. they continue onto the valve mantle, whereas in *L. ventricosa* they terminate on the valve face (Van de Vijver et al. 2025).

**Table 1.** Comparison of basic morphological features of the taxa belonging to *Luticola ventricosa* complex.

Species	Shape	Size L/W [µm]	Striae [in 10 µm]	Areole per striae	Proximal raphe endings	Distal raphe endings	Reference
***L. bilyi***	linear, paralel margins, ± constricted	14.0–20.0/ 4.5–6.0	18–20	3–4	short, straight	short, straight	Levkov et al. 2013
***L. borealis* sp. nov**.	linear-elliptic, parallel margins	(9.0)12.5–28.0/6.0–8.5	16–21	4	unilaterally bent	hooked, continued onto valve mantle	this study
***L. dubia***	linear-lanceolate, valve margin convex	12.0–21.0/ 5.0–6.5	20–24	3–4	unilaterally bent	hooked, terminating on valve face	this study
***L. exilissima* sp. nov**.	linear-elliptic, parallel margins	15.0–18.5/ 5.0–6.0	20–22	3–4	tiny, sharply hooked with small depression	hooked, continued onto valve mantle	this study
***L. jagodae* sp. nov**.	narrow, linear-lanceolate	10.0–25.0/ 4.0–6.0	17–21	3	curved	hooked, terminating on valve face/mantle junction	this study
***L. lacunicola* sp. nov**.	linear-elliptic, paralel margins	15.0–19.0/ 5.5–6.0	18–22	4–5	almost straight	strongly bent, terminating on valve face	this study
***L. levkovii***	linear-lanceolate to linear-elliptic	13.0–31.5/ 5.8–7.8	17–20	(4)5–6	curved	hooked, continued onto valve mantle	this study
***L. levkovii***	linear-lanceolate to linear-elliptic	17.0–28.0/ 8.5–9.5	20–21	5–6	curved	hooked, continued onto valve mantle	Reichardt 2018
***L. speluncae* sp. nov**.	narrow, linear-lanceolate	16.0–20.0/ 5.0–5.5	16–18	3	unilaterally bent	deflected, terminating on valve face	this study
***L. microventricosa* sp. nov**.	elliptic	9.0–15.0/ 4.5–5.5	16–18	3	deflected	hooked, terminating on valve face	this study
***L. ventriconfusa***	linear-lanceolate, weakly undulate	10.0–19.0/ 5.8–6.8	20–24	4–5	bent with linear fissures	hooked, continued onto valve mantle	this study
***L. ventriconfusa***	linear-lanceolate, weakly undulate	10.0–22.0/ 6.0–8.0	20–24	4–5	slightly curved	hooked, continued onto valve mantle	Levkov et al. 2013
***L. ventricosa***	weakly asymmetrical, lanceolate-elliptic	12.0–18.0/ 5.5–6.5	19–22	(3)–4	unilaterally bent	hooked, terminating on valve face	Van de Vijver et al. 2025
***L. ventricosa***	lanceolate-elliptic with convex margins	10.0–20.0/ 4.5–6.5	17–23	(3)-4	unilaterally bent	hooked, terminating on valve face	this study

*Luticola lacunicola* sp. nov. can be readily distinguished from other species of the *Luticola ventricosa* complex by the position of the bucciniportula, which is slightly displaced towards the valve centre compared to other taxa in the group. The central area is small to very small, and is bordered by shortened striae composed manly by two areolae rather than single areolae, as observed in most similar taxa. Among morphologically similar taxa, *Luticola lacunicola* sp. nov. most closely resembles *Luticola exilissima* sp. nov., but both species can be reliably separated based on raphe morphology. In *Luticola lacunicola* sp. nov., the distal raphe endings terminate on the valve face, whereas in *L. exilissima* sp. nov. they extend onto the valve mantle. Furthermore, the proximal raphe endings in *Luticola lacunicola* sp. nov. are nearly straight, while those in *L. exilissima* sp. nov. are distinctly and strongly curved. *Luticola lacunicola* sp. nov. is most similar to *L. bilyi* in terms of valve dimensions and stria density. Valves of *L. lacunicola* sp. nov. measure 15.0–19.0 µm in length and 5.5–6.0 µm in width, with 18–22 striae in 10 µm, whereas *L. bilyi* is 14.0–20.0 µm long, 4.5–6.0 µm wide, and has 18–20 striae in 10 µm. The two taxa differ, however, in the number of areolae per stria, which is higher in *L. lacunicola* sp. nov. (4–5) compared to *L. bilyi* (3–4). In addition, valve outline clearly separates the two species: *L. lacunicola* sp. nov. has a linear-elliptic valve shape with parallel margins, whereas *L. bilyi* is linear with slightly constricted margins and proportionally larger apices relative to the valve width.

Both *Luticola borealis* sp. nov. and *L. levkovii* stand out within the *L. ventricosa* complex by generally more robust apices compared to the other species. Despite this similarity, the two taxa can be distinguished based on several characters. Valve dimensions of both species are comparable (see Table 1). Among them only number of areolae per striae seems to distinguish both taxa: *L. borealis* sp. nov. generally has four, whereas *L. levkovii* mainly exhibits five to six. Moreover, both taxa can be separated based on proximal raphe endings morphology, which are bent away from the bucciniportula in *L. borealis* sp. nov., contrasting with the clearly curved proximal endings observed in *L. levkovii* (Reichardt 2018).

Within the *Luticola ventricosa* complex, ecological preferences vary considerably, reflecting both true habitat diversity and historical misidentifications. *Luticola ventricosasensu stricto* has been reported from a wide range of habitats, including freshwater bodies, springs, soils, and terrestrial mosses; however, many of these records may pertain to other, previously unrecognized species. The populations examined in this study originated primarily from aerophytic and terrestrial environments, particularly mosses growing on various substrates and moisture levels, ranging from mosses on mill walls to mosses on tree trunks and decaying logs. Literature records also confirm the species’ ability to grow on soils (Foets et al. 2020). In contrast, *L. ventriconfusa* has been observed exclusively in aquatic habitats, and earlier soil records likely result from misapplication of the taxon name to other species within the *L. ventricosa* complex, most commonly *L. borealis* sp. nov. (Rybak, unpublished data; Stanek-Tarkowska, pers. comm.). *Luticola dubia*, originally described from halomorphic soils in Macedonia (Levkov et al. 2017), has not been recorded more broadly to date – which may indicate both a limited distribution and narrow ecological preferences of the species. Ecological preferences for aerophytic conditions similar to those observed in *L. ventricosasensu stricto* are also likely exhibited by *L. speluncae* sp. nov. (found in moist mosses at the entrance to a cave), *L. microventricosa* sp. nov. (inhabiting wet mosses near the Crn Kamen River), and *L. exilissima* sp. nov. (collected from the surface of a damp rock). *Luticola jagodae* sp. nov., by contrast, was collected from dry mosses growing on soil near Lake Salda and at the base of a limestone rock at the same location, suggesting a more xerophilous ecology than the other species. *Luticola lacunicola* sp. nov. was observed in a few alpine ponds on Shara Mountain, where the type locality is a small and shallow pond that remains completely frozen from late autumn to late spring. Diatom flora at this site is quite diverse, including *L. scardica* Levkov, Metzeltin & A. Pavlov (Levkov et al. 2013) and several *Muelleria* (J.Frenguelli) J.Frenguelli species (Levkov et al. 2019). *Luticola bilyi* was always found rarely and exclusively in mountain habitats (Levkov et al. 2013). *Luticola levkovii* occurs in small ponds, such as in Treuchtlingen, Germany (Reichardt 2018), as well as in North Macedonia in temporary wet ponds formed by springs on Osogovo Mountain (Levkov et al. 2025). Among all species, *Luticola borealis* sp. nov. appears to have the broadest ecological preferences, having been observed on soils (Stanek-Tarkowska, pers. comm.), mosses on tree bark (Rybak et al. 2023; reported as *L. ventricosa* MT1), highly saline puddles (Rybak et al. 2019; reported as *L. ventricosa* MT1), and various freshwater bodies like: oligotrophic waters at higher altitude (South-eastern Europe) or streams, ponds and peatbogs (this study; Wojtal 2009; Berezovska 2024). Additionally, this species also appears to have a very wide geographical distribution, which, combined with its noticeably different valve shape compared to *L. ventricosasensu stricto*, has in the past led to misidentifications as distinct taxa were commonly force fitted under different names (e.g., Wojtal 2009, pl. 72 fig. 3 – reported as *L. ventricosa*; Kulikovskiy et al. 2016, pl. 57, figs 13–15 – reported as *L. permuticopsis* Kopalová & Van de Vijver; Berezovska 2024, pl. 3 fig. 2 – reported as *L. muticopsis* (Van Heurck) D.G.Mann).

The detailed examination of type material and comparative analyses presented here have clarified the taxonomic boundaries within the *Luticola ventricosa* complex. Several previously unrecognized species have been distinguished. These findings underscore the importance of careful taxonomic revision for understanding both the distribution and the ecology of diatom species, and they provide a foundation for future biogeographical and ecological studies within the genus *Luticola* D.G.Mann.

## Supplementary Material

XML Treatment for Luticola ventricosa

XML Treatment for Luticola dubia

XML Treatment for Luticola levkovii

XML Treatment for Luticola ventriconfusa

XML Treatment for Luticola borealis

XML Treatment for Luticola exilissima

XML Treatment for Luticola jagodae

XML Treatment for Luticola lacunicola

XML Treatment for Luticola microventricosa

XML Treatment for Luticola speluncae

## Additional information

### Artificial Intelligence (AI) use

The authors accept full responsibility for the content of the manuscript, including the disclosure of any use of AI.

No AI tools were used in the preparation of this manuscript.

### Author ORCIDs

M. Rybak https://orcid.org/0000-0001-8998-9537

Z. Levkov https://orcid.org/0000-0002-1184-2356

### Data availability

All of the data that support the findings of this study are available in the main text.
